# PEGylated Domain I of Beta-2-Glycoprotein I Inhibits Thrombosis in a Chronic Mouse Model of the Antiphospholipid Syndrome

**DOI:** 10.3389/fimmu.2022.842923

**Published:** 2022-04-11

**Authors:** Rohan Willis, Thomas C. R. McDonnell, Charis Pericleous, Emilio B. Gonzalez, Alvaro Schleh, Zurina Romay-Penabad, Ian P. Giles, Anisur Rahman

**Affiliations:** ^1^Internal Medicine, University of Texas Medical Branch, Galveston, TX, United States; ^2^Centre for Rheumatology Research, Division of Medicine, University College London, London, United Kingdom

**Keywords:** antiphospholipid syndrome, beta-2-glycoprotein I, PEGylation, domain I, thrombosis

## Abstract

Antiphospholipid syndrome (APS) is an autoimmune disorder in which autoantibodies cause clinical effects of vascular thrombosis and pregnancy morbidity. The only evidence-based treatments are anticoagulant medications such as warfarin and heparin. These medications have a number of disadvantages, notably risk of haemorrhage. Therefore, there is a pressing need to develop new, more focused treatments that target the actual pathogenic disease process in APS. The pathogenic antibodies exert their effects by interacting with phospholipid-binding proteins, of which the most important is beta-2-glycoprotein I. This protein has five domains, of which the N-terminal Domain I (DI) is the main site for binding of pathogenic autoantibodies. We previously demonstrated bacterial expression of human DI and showed that this product could inhibit the ability of IgG from patients with APS (APS-IgG) to promote thrombosis in a mouse model. Since DI is a small 7kDa protein, its serum half-life would be too short to be therapeutically useful. We therefore used site-specific chemical addition of polyethylene glycol (PEG) to produce a larger variant of DI (PEG-DI) and showed that PEG-DI was equally effective as the non-PEGylated DI in inhibiting thrombosis caused by passive transfer of APS-IgG in mice. In this paper, we have used a mouse model that reflects human APS much more closely than the passive transfer of APS-IgG. In this model, the mice are immunized with human beta-2-glycoprotein I and develop endogenous anti-beta-2-glycoprotein I antibodies. When submitted to a pinch stimulus at the femoral vein, these mice develop clots. Our results show that PEG-DI inhibits production of thromboses in this model and also reduces expression of tissue factor in the aortas of the mice. No toxicity was seen in mice that received PEG-DI. Therefore, these results provide further evidence supporting possible efficacy of PEG-DI as a potential treatment for APS.

## Introduction

Antiphospholipid syndrome (APS) is an autoimmune disease in which autoantibodies cause clinical features of arterial or venous thrombosis or pregnancy morbidity. The pathogenic antibodies in APS are generally termed antiphospholipid antibodies (aPL) although they generally bind phospholipid-protein complexes. APS has a population prevalence of approximately 1 in 2000 (1) and can be diagnosed where a patient has at least one clinical feature (thrombosis or pregnancy morbidity) together with persistent positivity in at least one of the three serological assays for aPL that were cited in the most recent classification criteria for APS (2) and are in routine clinical use. These assays are the anti-cardiolipin (aCL) ELISA, the lupus anticoagulant assay and the anti-beta-2-glycoprotein I ELISA. Beta-2-glycoprotein I is present in a concentration of 200mcg/ml in human serum and has a wide range of biological functions including roles in both the complement and coagulation cascades (3). Pathogenic antibodies in APS primarily bind the N-terminal domain (Domain I) of beta-2-glycoprotein I (4, 5). This leads to the formation of trimeric complexes comprising one antibody molecule and two beta-2-glycoprotein I molecules. These complexes interact with anionic phospholipids and membrane receptors in the surface membranes of target cells such as monocytes, endothelial cells and platelets. The interaction stimulates change in cellular behavior such as release of tissue factor (TF), thus leading to the clinical features of the disease (6).

Current therapeutic options for APS are limited. The only evidence-based treatment to prevent recurrent thrombosis is long-term anticoagulation (7–9). This is usually achieved by prescribing vitamin K antagonists such as warfarin with disadvantages including the need for regular monitoring of blood tests and risk of haemorrhage. Although it was hoped that the introduction of direct acting oral anticoagulants such as rivaroxaban would reduce reliance on warfarin in patients with APS (10), recent trials have not favored this option (11, 12). In patients who test positive for aPL, but who have not yet suffered thrombosis, there is no strong evidence base for any treatment to protect against the first thrombotic event, though aspirin can be used in patients with a particularly high risk profile (9, 13).

It is therefore important to develop new forms of therapy for APS (12) which, rather than causing non-specific anticoagulation, block the pathogenesis of the syndrome in a more specific manner. One possibility is to block binding of pathogenic aPL to Domain I of beta-2-glycoprotein I (DI). Previously we developed a bacterial expression system for DI (14) and showed that the recombinant protein produced was able to block thrombosis induced by passive administration of IgG purified from serum of patients with APS (APS-IgG) in a mouse model (15).

DI is a small 7kDa peptide and therefore unsuitable for therapeutic use because its serum half-life would be very short. A number of techniques have been used to increase the size of such molecules to improve their therapeutic potential. These include addition of the immunoglobulin Fc region as in the anti-tumor necrosis drug etanercept and chemical addition of a polyethylene glycol (PEG) group. The latter process is called PEGylation and has been used in a number of drugs in current therapeutic use, notably pegloticase used in gout and certolizumab pegol used in rheumatoid arthritis [reviewed in (16)]. The size and method of attachment of the PEG group can both be modified. Larger PEG groups are beneficial in terms of increasing half-life and reducing immunogenicity but could potentially reduce biological activity by blocking interaction of the peptide with its biological ligand (17, 18). Whereas non-specific PEGylation of a peptide leads to a range of possible products with variable activity, site-specific PEGylation on disulfide bonds allows predictable and reproducible properties of the PEGylated product (19). However, development of anti-PEG antibodies can be a problem and has been described particularly for pegloticase, where it reduces the efficacy of the drug (20).

Since DI carries two disulfide bonds, it is suitable for site-specific PEGylation. In a previous issue of *Frontiers in Immunology*, we described site-specific PEGylation of DI and the purification and characterization of the PEGylated product (21). We showed that the PEG-DI obtained was able to inhibit binding of APS-IgG to whole beta-2-glycoprotein I, to inhibit the effect of APS-IgG on clotting of human blood and to inhibit the ability of passively transferred APS-IgG to cause thrombosis in a mouse model (21).

This passive transfer model, in which a relatively large amount of extraneous IgG is administered to a mouse acutely, is an imperfect model of APS because the antibodies are present persistently in patients with that disease. Therefore, Pierangeli and colleagues developed an alternative model in which mice are immunized with human beta-2-glycoprotein I and develop anti-beta-2-glycoprotein I antibodies (22). When the mice were submitted to a traumatic stimulus at the femoral vein, the size of the thrombus produced was significantly larger than in control mice immunized with human serum albumin.

In this paper, we investigate the inhibitory effects of PEG-DI on thrombosis in this chronic mouse model of APS.

## Methods

### Production of PEG-DI

Bacterial expression and PEGylation of DI were described fully in our previous paper in Frontiers in Immunology (21). In brief, *E. coli* BL21* cells are transfected with the recombinant DI plasmid and expression of DI is achieved by addition of 1 mM IPTG followed by incubation with shaking overnight at 20°C. The PEG-DI originally collects in inclusion bodies, which are solubilized in a chaotropic buffer by bacterial lysis, sonication and centrifugation followed by grinding using a mortar and pestle. The expressed protein carries an N-terminal hexahistidine tag such that it can be purified on a nickel column. The purified protein is re-folded in 0.6 M arginine buffer with a cysteine-cystine buffer (pH 8.5) and dialysed against 20 mM Tris, 0.1 M NaCl, pH 8. Protein is again purified post-folding using a nickel column and dialysed against phosphate buffered saline (PBS).

Protein was reduced at a concentration of 0.4 mg/ml in 2 M arginine, 20 mM sodium phosphate (NaPO4, 0.1 M NaCl), 40 mM EDTA at pH 8.0 with 0.1 M DTT for 1 h at 20°C. This process was followed by removal of the reductant and buffer exchange on a PD-10 column to an identical buffer with 25 mM arginine rather than 2 M. PEGylation reagent was added (1:0.8 molar ratio) and incubated for 4 h at 4°C. This solution was then buffer exchanged to 20 mM sodium acetate with 0.05% Tween at pH 6.0 for cation exchange purification on a 5 ml SP-HP column (GE Healthcare) with a linear gradient from 20% buffer containing 1 M NaCl to 100% of the same buffer at 2 ml/min for 1 h. Fractions containing protein of the expected size of PEG-DI were identified by peaks on a chromatogram at 280 nm and then pooled. The hexahistidine tag was cleaved using FXa as in McDonnell et al (23). This was followed by a single isocratic wash in SEC(16/600, Superdex 75) buffer. For this experiment two different versions of PEG-DI carrying 20kDa PEG and 40kDa PEG were prepared and their properties compared with non-PEG-DI. All preparations were incubated in an endotoxin removal column (Pierce High-Capacity Endotoxin Removal Resin, ThermoScientific) until found to be endotoxin free by the fluorescent endotoxin assay (Hyglos).

Both DI and PEG-DI have been shown to be biologically active in a range of assays, indicating that the expressed DI is correctly folded (4, 21).

### Preparation of Proteins β2GPI and OA for Immunization Protocol

β2GPI was isolated from pooled normal human serum, as described in detail elsewhere (24). In brief, human β2GPI was purified using perchloric acid precipitation and affinity chromatography on a heparin-sepharose column (HiTrap HP, GE Healthcare). The eluted material from this first step was then subjected to ion exchange chromatography on a Resource-S column (GE Healthcare). The purity of all β2GPI preparations was confirmed by SDS-PAGE (Mini-Protean TGX 4-20% gel, BioRad) and antigenicity determined by coating ionization-treated polystyrene plates and measuring binding to known anti-β2GPI patient sera in an ELISA procedure as described elsewhere (24). Purified ovalbumin (Sigma-Aldrich) was purchased. All preparations were treated until determined to be free of endotoxin contamination (< 1.0 EU/mL).

### Chronic Mouse Model of APS

The method was as described in previous papers (22). Male CD-1 mice (n=5 per group) (Charles River Laboratories, Wilmington, MA) between 3-4 weeks in age (10-15g) were immunized intraperitoneally (IP) with 3 consecutive weekly doses of 0.5 µg of β2GPI in sterile PBS with an equal volume of complete Freund’s adjuvant (CFA) at week 0 or incomplete Freund’s adjuvant (IFA) at weeks 1 and 2. Negative control mice were injected IP with 0.5 µg of ovalbumin (OA) in CFA or IFA over the 3-dose immunization regimen. The dose of 0.5 µg of β2GPI used in these experiments was based on our previous experiments to optimize the model. We have found that this dose leads to development of anti-β2GPI antibodies in the mice at levels giving optical density of 1.5 to 2.0 in ELISA, similar to results obtained from patients with APS.

Blood was collected retro-orbitally once weekly prior to treatment given at each time point to test development of antiphospholipid antibodies using in-house ELISA assays according to a standardized protocol as previously outlined (24). Terminal surgery to evaluate outcome measures was performed four weeks after initial immunization dose. At 72 hours prior to terminal surgery, a therapeutic dose (20*µg)* of DI peptide construct in sterile PBS or sterile PBS only as a control was given and a second similar dose of the same construct given at 24 hours prior to terminal surgery.

At week 4, mice were anaesthetised and one of the femoral veins was exposed for observation with an approximate 0.5mm segment trans-illuminated using a microscope equipped with a closed-circuit video system. The isolated vein segment was pinched to introduce a standardized injury and thrombus formation and dissolution was visualized and recorded. The treatment groups were as follows: ***1)*** β2GPI *Immunization/PBS treatment (positive control)*, ***2)*** *OA Immunization/PBS treatment (negative control)*, ***3)*** *β2GPI Immunization/20 µg non-PEG-DI*, ***4)*** β2GPI *Immunization/20 µg 20 kDa PEG-DI*, ***5)*** *β2GPI Immunization/20 µg 40 kDa PEG-DI.*

Three outcome measures were assessed, as fully described in previous papers with modifications as outlined below (15). These were:

a) Induced thrombus size: A total of three thrombi were generated in each mouse and the largest cross-sectional area of each thrombus during the formation-dissolution cycle was measured five times and a mean value calculated (in μm^2^). Each thrombus dissolves before the next is formed.
b) Tissue Factor (TF) expression in mouse aorta homogenates (measured as described in (b) above). At the end of surgery, mice were perfused with heparin and protease inhibitor cocktail solution and the descending aorta collected (from aortic arch to renal branches). Excess peri-aortic fat was removed and the aorta flash-frozen in heparin and protease inhibitor cocktail and stored at -70°C until processed for TF testing as previously described.
c) Activity of TF in peritoneal macrophages by a solid-phase immunoassay specifically designed to measure mouse TF (Picokine ELISA, Boster Biological). Results were standardized with reference to the protein concentration of lysates (BCA Protein assay, ThermoScientific Pierce) and expressed in pg/µgml^-1^ units.

All animals were housed in the viral antibody-free barrier facility at the University of Texas Medical Branch (UTMB). Animal use and care were in accordance with the UTMB Institutional Animal Care and Use Committee (IACUC) guidelines.

### Statistical Analysis

Results were expressed as means plus or minus standard deviation as appropriate. A one-way analysis of variance by ANOVA followed by the Tukey-multicomparison test was used to compare differences among mouse groups.

## Results

### Immunization of Mice With β2GPI Induces aPL Production

**Figure 1:**
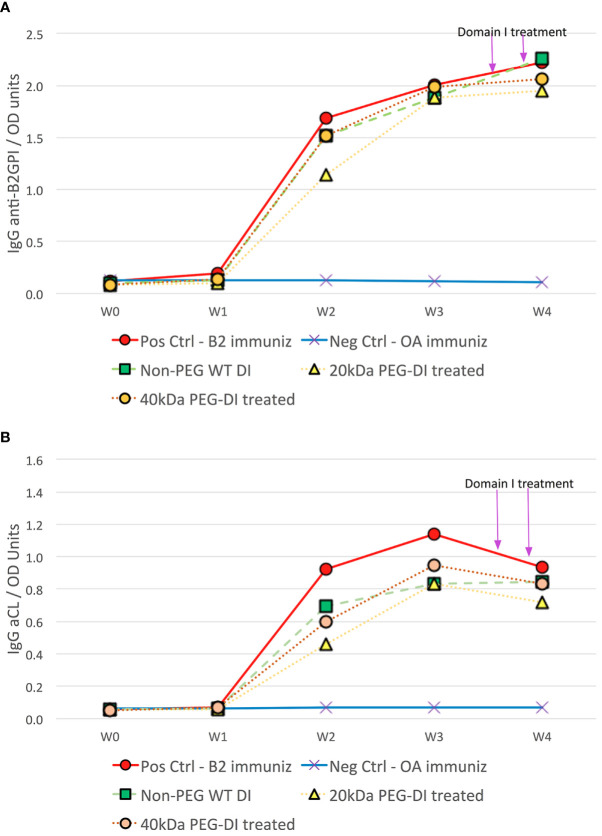
Antiphospholipid antibody development in chronic mouse model of APS disease. CD1 mice immunized with β_2_GPI produced significant titers of anti-β_2_GPI **(A)** and aCL **(B)** over the 4-week period compared to corresponding OA immunized negative controls. At the time of thrombus surgery at week 4, aCL and anti-β_2_GPI titers in positive control β_2_GPI-immunized mice were similar to those in β_2_GPI-immunized mice treated with non-PEG-DI, 20kDa PEG-DI and 40kDa PEG-DI prior to surgery.

Compared to controls treated with OA, β2GPI-immunized CD1 mice produced significantly greater mean anti-β2GPI levels by week 2 (1.14 ± 0.68 to 1.69 ± 0.39 vs-0.13 ± 0.01OD, p<0.0001) and continued to be significantly elevated over the remaining length of the 4-week test period (p<0.0001). Close to maximum anti-β2GPI levels were attained in week 3 (1.88 ± 0.43 to 2.00 ± 0.38 vs-0.12 ± 0.01OD, p<0.0001) and maintained in week 4 at the time of thrombosis surgery (1.95 ± 0.40 to 2.23 ± 0.24 vs-0.10 ± 0.01OD, p<0.0001). Mean levels of anti-β2GPI at the time of surgery were statistically similar in all groups of mice immunized with β2GPI (p=0.624) (**Figure 1A**).

The associated increase in aCL levels occurred in a similar fashion albeit less robustly. Mean aCL levels significantly increased by week 2 (0.46 ± 0.34 to 0.93 ± 0.61 vs-0.07 ± 0.01OD, p<0.0001) and continued to be significantly elevated over the remaining length of the 4-week test period (p<0.0001). Close to maximum aCL levels were attained in week 3 (0.83 ± 0.22 to 1.14 ± 0.38 vs 0.07 ± 0.01OD, p<0.0001) but waned slightly in week 4 at the time of thrombosis surgery (0.72 ± 0.27 to 0.93 ± 0.28 vs 0.07 ± 0.01OD, p<0.0001). Mean levels of aCL at the time of surgery were statistically similar in all groups of mice immunized with B2GPI (p=0.661) (**Figure 1B**).

### PEGylated DI Retains the Ability to Inhibit the Effect of Induced APS-IgG on Development of Thrombosis in a Chronic Mouse Model of APS

**Figure 2:**
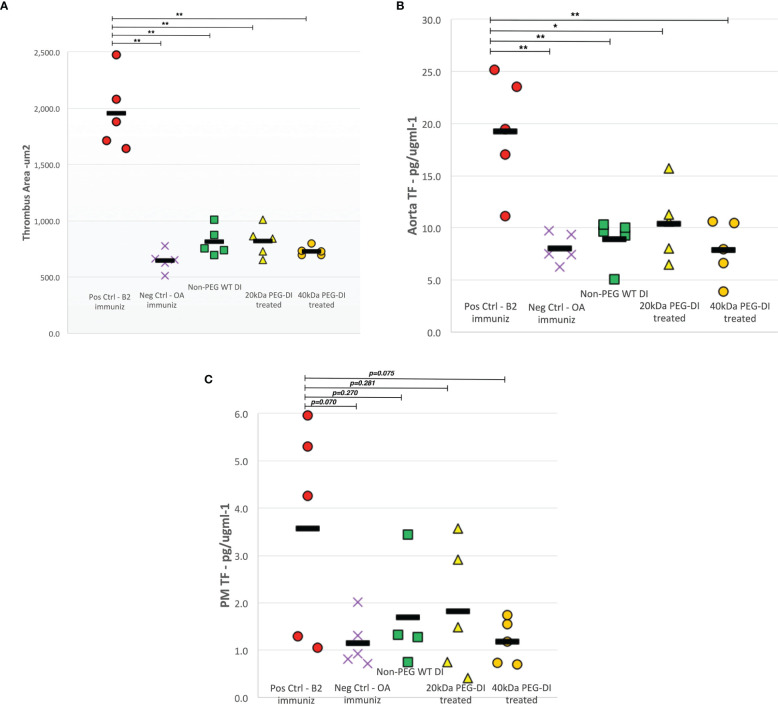
Thrombus area, aortic and peritoneal macrophage tissue factor levels in chronic APS mouse model. Both thrombus area **(A)** and aortic TF **(B)** levels decreased significantly in β_2_GPI-immunized mice treated with non-PEG-DI, 20kDa PEG-DI and 40kDa PEG-DI compared to β_2_GPI-immunized positive control mice. The abrogated levels of thrombus development and aortic TF were similar to that seen in negative control mice. While there was a trend of decreased peritoneal macrophage TF **(C)** levels in all domain I treated mice, the noted reductions were not statistically significant. (*p<0.01, **p<0.001).

In each panel of **Figure 2**, the two columns on the far left show the difference in outcome obtained after a 4-week immunization course with β2GPI (pos control) and OA (neg control) in the absence of any inhibitor. In **Figure 2A**, thrombus size was much larger in positive controls compared to negative controls (1958.3µm^2^ vs 645.7µm^2^, p<0.0001). Non–PEG-DI treatment significantly reduces thrombus size compared to positive control mice (815.3µm^2^, p<0.0001) to a size very similar to that obtained in negative controls (p=0.570). The following two columns show that 20kDa and 40kDa PEG-DI also inhibit thrombosis (818.9µm^2^, p<0.0001 and 728.9µm^2^, p<0.0001 respectively), with no noted loss of inhibitory activity compared to the non-pegylated construct (p=0.937)

**Figure 2B** shows TF expression in aorta homogenates of mice immunized with β2GPI and OA. A similar outcome profile was seen with significantly higher TF levels in β2GPI immunized mice, approximately 2.5 times that recorded in OA treated mice (19.3 ± 5.6 vs. 8.1 ± 1.4 pg/µgml^-1^, p=0.0003). Non-PEG-DI treatment abrogated TF expression in β2GPI immunized mice (p=0.001) to levels similar to control mice. Both the 20KDa and 40kDa PEG-DI also inhibited TF expression with no noted loss of inhibitory activity compared to the non-pegylated construct (p=0.951).

**Figure 2C** shows TF expression in peritoneal macrophages of immunized mice with a similar outcome profile. Higher TF levels were recorded in β2GPI immunized mice compared to OA treated mice and non-PEG-DI, 20kDa PEG-DI and 40kDa PEG-DI treatment decreased TF expression to levels seen in negative control mice. However, these changes only approached statistical significance (p=0.070).

There was no toxicity noted in any mice treated with the PEGylated or non-PEGylated construct treatments in this chronic active immunization mouse model. In previous experiments using a passive immunization model in which external immunoglobulins were administered, there were noted adverse reactions. These were completely absent in this disease model in which mouse autoantibodies were induced to simulate active APS disease.

## Discussion

In this paper, we have built on our previous paper describing the efficacy of PEG-DI in the passive immunization model (21). In that paper, we tested both wild-type (WT) DI and an altered variant containing two point mutations at positions 8 and 9 that we thought might have better inhibitory properties than the wild-type form based on previous experiments on non-PEGylated DI (15). In the event, however, the PEGylated version of this DI variant was not more effective and also had a significant toxicity problem with 40% of mice treated dying. Therefore, in the current experiment, only WT-DI was tested. The other important difference between this paper and the previous one (21) was our use of the chronic mouse APS model (22). Patients with APS have chronically elevated levels of aPL, with higher levels of aPL and triple-positivity for aCL, anti- β2GPI and lupus anticoagulant both being associated with higher risk of thrombosis (25). The presence of these antibodies, however, does not in itself lead to thrombosis. Some patients may be triple-positive for many years without developing thrombosis. This leads to the concept that a “second hit” is required by which some other stimulus such as pregnancy or surgery leads to a new thrombosis in a person predisposed to that by the presence of serum aPL. This chronic mouse model recapitulates what is seen in patients. As shown by our results, immunization with β2GPI leads to persistently elevated levels of aCL and anti- β2GPI in the mice but they do not develop thrombosis till subjected to the femoral vein trauma. It is therefore very encouraging that PEG-DI was just as effective as non-PEG-DI in inhibiting thrombosis in this model and caused no toxicity. As opposed to the passive transfer model that we used previously, the chronic model has a number of advantages including the potential to recapitulate processes such as B and T cell clonal expansion, epitope spreading and break in tolerance. For example, we previously used this model to demonstrate both T cell-dependent and independent aPL production (24).

Although there have been previous attempts to develop therapies for APS based on blocking intermolecular interactions of β2GPI with either antibodies or phospholipids, they have not focused on DI. Whereas DI is the main site of binding for pathogenic anti-β2GPI antibodies, the C-terminal DV interacts with anionic phospholipids on cell surfaces *via* a lysine-rich patch on the domain (26). It is believed that this interaction of surface phospholipids with the antibody- β2GPI complex leads to cellular effects critical to the pathogenesis of APS (6). Therefore, several groups have attempted to block the phospholipid-DV interaction rather than the antibody-DI interaction in developing new therapies for APS.

TIFI is a 20 amino acid peptide that has homology to DV and inhibits the binding of DV to phospholipids. Vega-Ostertag and colleagues demonstrated that TIFI blocked binding of β2GPI to human umbilical vein endothelial cells *in-vitro* and also inhibited thrombosis caused by passive administration of IgG from patients with APS in the same model that we previously used (27). A control peptide VITT (containing similar amino acids to TIFI but in a different order) did not inhibit thrombosis. A different group, in Italy, studied the efficacy of TIFI in a mouse model of obstetric APS. They showed that TIFI inhibited binding of APS-IgG to human trophoblast cells whereas VITT did not (28) Furthermore, when pregnant C57Bl6 mice were treated intravenously with either monoclonal human aPL or control IgG from healthy people, only the aPL-treated mice suffered fetal loss and reduced fetal and placental weight. All these pathogenic effects of aPL were reduced by treatment with TIFI at days 0,5 and 10 before sacrifice at Day 15 whereas they were not reduced by VITT (28). TIFI, however, is a small molecule that would suffer the same problem of short half-life that we have described for native DI. No-one has yet published any data on forms of TIFI that have been modified to increase serum half-life.

An alternative approach has been described in a series of papers from the group of Kolyada and colleagues (29–32). They have taken advantage of the fact that the A1 domain of the apolipoprotein E receptor 2 (ApoER2) binds to DV. By creating a dimer of A1 in which two A1 molecules are joined by a flexible linker, they created an agent that blocked binding of β2GPI-anti β2GPI complexes but not of non-complexed β2GPI to CL (29). They then showed that the same agent reduced thrombosis induced by a laser in both an autoimmune (NZWxBXSB1)F1 mouse model of APS and in healthy strain mice that had been infused passively with APS-IgG (30). They also showed that a point mutant of A1-A1 dimer had better inhibitory properties *in-vitro* than wild-type A1-A1 (31). The A1-A1 dimer, however, has the same problem of short serum half-life that pertains to DI and TIFI. In the mouse model experiments, 84% of A1-A1 had been lost from the blood within an hour (30). In a subsequent experiment in (NZWxBXSB1)F1 mice, this problem was counteracted by use of a subcutaneous osmotic pump to deliver the agent continuously over 2-4 weeks (32). A reduction in the blood pressure of the mice was reported, but thrombosis was not an outcome of this study (32). It is unlikely that patients would accept use of such a device for a treatment that is essentially preventative, and there are no reports of chemical modification of A1-A1 to increase serum half-life.

In conclusion, there is a pressing need for new therapies for APS. The current standard of care with vitamin K antagonist anticoagulants such as warfarin is fraught with difficulties such as risk of haemorrhage. The initial promise of direct oral anticoagulants has unfortunately not been fulfilled. Therapies that target the interaction of DV of β2GPI with phospholipids have shown encouraging results in mice, but none have been modified to give serum half-life that would allow therapeutic use in patients. Our work in developing a PEGylated inhibitor of the anti- β2GPI-DI interaction is therefore important and we have now shown its efficacy in both a chronic autoimmune and an acute passive transfer model of APS. The mechanism of the effect is not yet clear and it remains to be demonstrated whether PEG-DI alters production or serum levels of anti-DI antibodies in these mice.

It is important to recognize that up to 20% of anti- β2GPI antibodies in patients with APS bind epitopes outside DI and so PEG-DI will not be effective in all patients. We have not yet shown an improvement in serum half-life of the PEGylated variant, though in the previous experiment (21) the inhibitory effects of the agent were demonstrable 48 hours after the last dose of PEGylated DI. Formal pharmacokinetic and toxicology experiments will now be important.

## Data Availability Statement

The raw data supporting the conclusions of this article will be made available by the authors, without undue reservation.

## Ethics Statement

The animal study was reviewed and approved by University of Texas Medical Branch Institutional Animal Care and Use Committee.

## Author Contributions

TM carried out laboratory work, developed methodology and produced PEG-DI. RW carried out mouse work in Texas and co-wrote the paper. CP developed methodology and gave intellectual guidance. IG gave intellectual guidance. EG and AS gave direction and support regarding experiments in Texas. ZR-P carried out practical work in Texas regarding mouse models. AR gave intellectual input, co-wrote the paper and supervised the project. All authors contributed to the article and approved the submitted version.

## Funding

Funding in Texas was from NIH grants: UL1TR001439 and R01AR56745. Funding at UCL included an MRC Development Pathway Funding Scheme Grant MR/P017371/1 and an NIHR research grant: RCF199, supported by the National Institute for Health Research University College London Hospitals Biomedical Research Centre. The authors thank PolyTherics/Abzena Limited for use of their proprietary method of PEGylation.

## Conflict of Interest

TM, CP, IG, and AR are all named co-inventors on a patent filed in the US for PEG-DI.

The remaining authors declare that the research was conducted in the absence of any commercial or financial relationships that could be construed as a potential conflict of interest.

## Publisher’s Note

All claims expressed in this article are solely those of the authors and do not necessarily represent those of their affiliated organizations, or those of the publisher, the editors and the reviewers. Any product that may be evaluated in this article, or claim that may be made by its manufacturer, is not guaranteed or endorsed by the publisher.

## Abbreviations

APS, Antiphospholipid syndrome; PEG, Polyethylene glycol; DI, Domain I of beta-2-glycoprotein I; TF, Tissue factor; CL, cardiolipin; *β*2GPI, beta-2-glycoprotein I.
